# Bridging eastern and western personality frameworks: a network approach integrating the Quantum-8-personality and Big Five personality traits

**DOI:** 10.3389/fpsyg.2026.1704706

**Published:** 2026-10-02

**Authors:** Qinying Lin, Yujie Ma, Youcheng Ma, Liangsong Li

**Affiliations:** 1 School of Chinese Medicine, Beijing University of Chinese Medicine, Beijing, China; 2 School of Public Health, Peking University, Beijing, China; 3 Shenzhen Hospital, Beijing University of Chinese Medicine, Shenzhen, China

**Keywords:** Big Five personality, cultural differences, network analysis, personality assessment, Quantum-8-personality

## Abstract

**Background:**

The Big Five Personality (BFP) model is an established dimensional framework, whereas the Quantum-8-Personality (Q8P) model is an emerging culturally grounded framework derived from Chinese philosophical traditions. This study explored dimension-level relationships between the two frameworks.

**Methods:**

Network analysis was conducted using data from 1,052 Chinese adults who completed the Quantum-8-Personality Scale (Q8PS) and Big Five Inventory-2 (BFI-2).

**Results:**

The largest cross-framework connections involved Q8P Contradiction and BFP neuroticism, Q8P Extraversion and BFP extraversion, and Q8P Yanggang and BFP conscientiousness. These associations were small to moderate in magnitude.

**Conclusion:**

The findings identify preliminary areas of correspondence between the Q8P and BFP dimensions but do not establish equivalence, convergent validity, or incremental validity of the Q8PS. The results should be interpreted as exploratory because the factorial validity of the Q8PS remains provisional.

## 1 Introduction

Personality is the characteristic pattern of cognition, emotion, and behavior that differentiates individuals. It reflects a person’s relatively stable tendencies across situations and is a foundational element of psychological functioning (Corr and Matthews, 2020). Although personality traits exhibit considerable consistency over time, they are not entirely stable. They evolve in response to psychological maturation, life experiences, and social context (Hudson, 2021).

Personality is associated with social functioning and behavior across multiple areas of life. Meta-analyses have documented relationships between personality traits and physical and mental health, academic performance, and occupational outcomes (Terracciano et al., 2025; Sharifibastan et al., 2025; Du et al., 2025; Han et al., 2024; Chen et al., 2025; Lee et al., 2024). For example, conscientiousness is often associated with healthier behavior and stronger occupational performance, whereas neuroticism is associated with greater vulnerability to anxiety and stress-related problems. These findings illustrate the practical relevance of studying personality while not implying that any single framework provides a complete account.

The exploration of own and others’ personality has long been a central theme in psychological research. Multiple personality assessment methods exist, ranging from projective techniques and behavioral observations to structured interviews. Nevertheless, owing to its efficiency and scalability, self-report questionnaire is the most widely used approach. Several prominent models have been used in contemporary personality studies. These include the Big Five Personality Traits (also known as the Five-Factor Model), Myers–Briggs Type Indicator (MBTI), Eysenck’s Psychoticism, Extraversion, and Neuroticism (PEN) Model, HEXACO Personality Model, 16 Personality Factors (16PF), and Dark Triad (encompassing narcissism, Machiavellianism, and psychopathy). Each offers a distinct theoretical lens to analyze individual personality differences (Table 1).

**Table 1:** The main points of various personality assessment tools.

Personality assessment tools	Authors	Personality model	Features
The Big Five personality traits	McCrae and John (1992)	Extraversion, Agreeableness, Conscientiousness, Neuroticism Openness	A comprehensive and widely validated framework with extensive empirical support.
Myers-Briggs Type Indicator (MBTI)	Myers et al. (1985)	16 different personality types based on four dichotomies: Extraversion vs. Introversion, Sensing vs. Intuition, Thinking vs. Feeling, Judging vs. Perceiving	Popular in applied settings; criticized for binary typology and limited empirical and cross-cultural validity (McCrae and Costa, 2004; Pittenger, 2005)
PEN Model	Airhihenbuwa (1990)	Psychoticism (P), Extraversion (E), Neuroticism (N)	Psychoticism is less empirically robust compared to Extraversion and Neuroticism (Padrell et al., 2023).
HEXACO Personality Model	Lee and Ashton (2004) and Ashton and Lee (2009)	This six-dimensional framework adds the Honesty-Humility dimension to the traditional Five-Factor Model.	Extends the Big Five with a sixth dimension; demonstrates strong applicability and predictive validity.
16 Personality Factor (16PF)	Cattell (1945)	16 primary personality traits.	Historically influential but less commonly used in contemporary research compared to other models.
Dark triad	Jonason and Webster (2010)	Narcissism Machiavellianism Psychopathy	Focuses on maladaptive and socially aversive personality traits.

Of these, the Big Five Personality (BFP) has garnered extensive empirical support and academic attention. As of August 19, 2026, it has been cited over 2,650,000 times according to Google Scholar, reflecting its dominant role in the field. The model conceptualizes personality along five broad dimensions: openness, conscientiousness, extraversion, agreeableness, and neuroticism. The HEXACO model builds upon this foundation by introducing a sixth dimension—honesty-humility—which captures variations in sincerity, fairness, and greed avoidance. In contrast, other models such as the PEN, 16PF, and Dark Triad have received comparatively less focus in recent research, although they are influential in specific applied contexts.

Culture is a crucial consideration in personality assessments. Cultural values, beliefs, and social norms shape the expression and development of personality traits, implying that instruments developed in one cultural context may not be fully appropriate in another (Bar and Otterbring, 2021; Allik and McCrae, 2004).

China, with its 5,000-year history of continuous civilization and rich philosophical heritage, presents a distinct cultural landscape. Many personality assessments used in China have been adapted from Western scales such as the Big Five Inventory-2 (BFI-2) and the MBTI. Although these adaptations provide valuable insights, they may have overlooked culturally specific constructs.

Notable exceptions exist in this regard. For example, the Five Pattern Scale, derived from the theory of traditional Chinese medicine, interprets personality through the lens of Wu Xing (the five elements)—metal, wood, water, fire, and earth—each associated with specific emotional and behavioral tendencies (Zhang et al., 2019). Similarly, the Quantum-8-Personality (Q8P) model is rooted in Chinese cultural philosophy, specifically the *I Ching* (Li, 2018) (Table 2).

**Table 2:** Quantum-8-personality and BaGua.

Trigram	Symbol	Five elements	Personality trait	Key features
Qián (Heaven)	☰	Metal	Yanggang	Steady, resilient, leadership
Kūn (Earth)	☷	Earth	Yinrou	Soft, introverted, nurturing
Duì (Lake)	☱	Metal	Introvertion	Shy, reserved, few words
Zhèn (Thunder)	☳	Wood	Extraversion	Impulsive, expressive, sociable
Xùn (Wind)	☴	Wood	Sensitivity	Perceptive, sentimental, detail-oriented
Gèn (Mountain)	☶	Earth	Deliberation	Honest, deliberate, steady
Lí (Fire)	☲	Fire	Contradiction	Indecisive, conflicted, emotionally complex
Kǎn (Water)	☵	Water	Harmoniousness	High emotional intelligence, adaptive

The I Ching is an ancient Chinese philosophical and divination system that has influenced East Asian thought for millennia (TenHouten and Wang, 2001). Its core components, the eight trigrams (BaGua), symbolize the dynamic and interdependent interplay between yin (represented by a broken line and associated with receptivity and negativity) and yang (unbroken line and activity and positivity). This system posits that all natural and human phenomena are results of a continuous transformation and balance between these two fundamental forces (Pham et al., 2006). Each trigram consists of three Yao (lines), and each Yao embodies both yin and yang qualities, reflecting the philosophy of duality and perpetual change (Table 2).

The Q8P model translates the ancient symbolism into a modern personality framework. The Q8P Scale (Q8PS) operationalizes this model with items largely derived from the theoretical work of Li (2018) and Ma (2024).

It proposes eight primary personality types, each corresponding to one of the trigrams and encompassing a unique combination of cognitive, emotional, and behavioral attributes rooted in the I Ching wisdom. Though eight-factor structure was not confirmed in an independent sample and that the scale should be regarded as provisional, this approach offers a culturally grounded alternative for understanding personality in the Chinese philosophical context.

The Q8PS exhibits the following key characteristics:

1. Incorporates dimensions such as Yinrou, Introversion, Sensitivity, Deliberation, and Harmoniousness, which are distinct from the Big Five BFP facets.
2. Employs culturally resonant representative figures to improve participant engagement and comprehension, offering an innovative approach for future AI-based personality assessments (Evin et al., 2022).
3. It is highly practical, with an average completion time of 3 min (compared to 6 min for BFI-2; Zhang et al., 2022), and a response rate of 99–100% for 40 items.
4. Uses a straightforward and interpretable scoring system. First, one personality corresponds to five items, and dimension scores are computed by summing the five corresponding item score. Second, the dimension with the highest score indicates the dominant personality type of the test-taker. Third, the evaluation incorporates overall score profiles to provide a comprehensive personality trait assessment. For instance, high scores on both Sensitivity and Contradiction dimensions may suggest a strong ability to perceive information, coupled with a limited “memory” capacity that impedes complex information processing; potentially leading to indecisiveness, internal conflict, and emotional behavior.

As a newly developed instrument, the Q8PS requires further validation. This study aimed to investigate the relationships between the Q8P and BFP dimensions through network analysis, facilitating cross-cultural integration and providing additional theoretical and methodological tools for personality research.

The two instruments differ in origin, validation status, and intended use. The BFI-2 is an established dimensional trait inventory supported by large-sample psychometric evaluation, including validation in Chinese populations (Zhang et al., 2022), and is used to compare broad personality domains. In contrast, the Q8PS is an emerging theory-derived descriptive instrument intended to characterize eight culturally grounded personality tendencies. It has not been validated for clinical diagnosis, personnel selection, nationally normed individual decisions, or use as a substitute for the BFI-2. Accordingly, the present study does not assume that the two instruments are equivalent or interchangeable; rather, it uses network analysis to examine exploratory dimension-level correspondence in Chinese adults while recognizing the provisional validation status of the Q8PS.

Within the Q8PS, the eight constructs are conceptualized as broad, relatively enduring, cross-situational tendencies spanning cognition, affect, interpersonal behavior, and behavioral style rather than as isolated actions, values, or abilities. Yanggang emphasizes persistence, responsibility, and assertive leadership; Yinrou emphasizes receptivity and nurturance; Introvertion emphasizes restraint and low outward expressiveness; Extraversion emphasizes social expressiveness; Sensitivity emphasizes rapid perceptual and affective responsiveness; Deliberation emphasizes a slow, cautious response style; Contradiction emphasizes ambivalence and internal conflict; and Harmoniousness emphasizes interpersonal attunement and accommodation. The eight scores are treated as correlated, nonorthogonal profile dimensions; the current model does not specify a higher-order hierarchy, and the highest score is descriptive rather than a mutually exclusive personality category. Conceptual overlap with the Big Five, HEXACO, interpersonal models, or trait emotional intelligence remains an empirical question requiring discriminant and incremental validation. Representative figures are used only as illustrations and are not part of scoring or validation.

## 2 Materials and methods

### 2.1 Participants

Participants were recruited using nonprobability convenience sampling. Trained university student investigators recruited adults through their social networks in their home provinces. Participants completed the Q8PS and BFI-2 as self-report measures in the same survey session.

The participants were 1,052 adults from various provinces (men: *n* = 412; women: *n* = 640; mean age = 26.18 years; SD = 11.2; range: 18–55 years). Confidentiality was assured, and participation was voluntary (Table 3).

**Table 3:** Basic characteristics of the population.

Characteristic		*n* (%)
Overall	Total sample	1,052 (100.00)
Age group	<35 years	803 (76.33)
	≥35 years	244 (23.19)
	Missing	5 (0.48)
Sex	Male	409 (38.88)
	Female	640 (60.84)
	Missing	3 (0.29)
Occupation	Student	676 (64.26)
	Management personnel	120 (11.41)
	Teacher	22 (2.09)
	Other occupation	234 (22.24)
Education	Primary school or below	8 (0.76)
	Junior high school	26 (2.47)
	Senior high school	93 (8.84)
	Bachelor’s degree	786 (74.71)
	Graduate degree	135 (12.83)
	Other	4 (0.38)
Province-level residence	Guangdong	389 (36.98)
	Beijing	150 (14.26)
	Yunnan	100 (9.51)
	Hubei	81 (7.70)
	Qinghai	78 (7.41)
	Zhejiang	63 (5.99)
	Guangxi	29 (2.76)
	Henan	22 (2.09)
	Jiangxi	13 (1.24)
	Ningxia	10 (0.95)
	Shanxi	10 (0.95)
	Shandong	9 (0.86)
	Guizhou	8 (0.76)
	Jiangsu	7 (0.67)
	Hebei	7 (0.67)
	Heilongjiang	7 (0.67)
	Taiwan	6 (0.57)
	Sichuan	6 (0.57)
	Inner Mongolia	5 (0.48)
	Hunan	5 (0.48)
	Fujian	5 (0.48)
	Xinjiang	4 (0.38)
	Shanghai	3 (0.29)
	Anhui	3 (0.29)
	Hainan	3 (0.29)
	Gansu	3 (0.29)
	Shaanxi	3 (0.29)
	Jilin	2 (0.19)
	Tianjin	2 (0.19)
	Hong Kong	2 (0.19)
	Macao	1 (0.10)
	Tibet	1 (0.10)
	Liaoning	1 (0.10)
	Chongqing	1 (0.10)
	Missing	13 (1.24)

The analytic sample was predominantly young and highly educated: 676 participants (64.3%) were students, 786 (74.7%) reported a bachelor’s degree, and 135 (12.8%) reported postgraduate education. The largest province-level groups were Guangdong (*n* = 389, 37.0%), Beijing (*n* = 150, 14.3%), and Yunnan (*n* = 100, 9.5%); the complete distribution is reported in Table 3. All participants were recruited through the same general investigator-mediated social-network approach, but investigator-specific recruitment proportions were not retained. Because recruitment was nonprobability-based and many province-level cells were small, formal regional comparisons were not used to make population-representative claims.

The inclusion criteria were as follows: (1) age: 18–55 years; (2) education level of junior high school or above, with the ability to comprehend the scale’s content; and (3) no history of depression or mental illness self-reported.

### 2.2 Personality assessments

#### 2.2.1 Quantum-8-personality scale (Q8PS)

The Q8PS was developed to operationalize eight personality constructs derived from traditional Chinese medicine mind–nature theory (Li, 2018; Ma, 2024): Yanggang, Yinrou, Introvertion, Extraversion, Sensitivity, Deliberation, Contradiction, and Harmoniousness. The item content was derived primarily from the theoretical descriptions of these eight constructs.

According to the development information available to the present authors, the developers first constructed an item pool and administered a preliminary version of the Q8PS to college students. Exploratory factor analysis was then used to screen the items, and items with primary factor loadings below 0.40 were removed. Participants were subsequently invited to comment on item comprehension and acceptability. Wording considered unclear or potentially uncomfortable was revised without intentionally changing the construct represented by the item. This process resulted in the current 40-item version of the Q8PS.

The available historical records did not contain the exact number of items in the original item pool, the sample size and demographic characteristics of the preliminary administration, the factor-extraction and rotation procedures used during the original item-screening analysis, the complete factor-loading and cross-loading matrix, or any prespecified item-deletion criteria beyond the loading threshold of 0.40. We therefore do not retrospectively infer or report procedures that could not be verified from the original development records. These limitations in the documentation of the initial scale-development process should be considered when evaluating the instrument.

To provide additional and reproducible psychometric evidence for the fixed 40-item version, we conducted a new exploratory factor analysis in the present sample of 1,052 adults from 25 Chinese provinces. The sample comprised 412 men and 640 women, with a mean age of 26.18 years (SD = 11.20; range = 18–55 years). All 40 items were complete and were treated as six-category ordered variables. The EFA was based on a polychoric correlation matrix. Sampling adequacy was acceptable (KMO = 0.881; minimum item MSA = 0.729), and Bartlett’s test of sphericity was significant, χ^2^(780) = 17,430.67, *p* < 0.001.

Factors were extracted using minimum residual estimation and direct oblimin rotation. Factor retention was evaluated primarily through parallel analysis using 500 simulated datasets, supplemented by the original and revised Velicer MAP procedures, the Kaiser criterion, comparisons of six- through nine-factor solutions, theoretical interpretability, and model parsimony. Parallel analysis supported eight factors, whereas the MAP procedures suggested six factors and the Kaiser criterion suggested nine. The eight-factor solution was retained for interpretation because it was supported by the primary empirical criterion and corresponded to the prespecified theoretical framework. This analysis evaluated the already finalized 40-item scale; it was not used to remove additional items.

The eight-factor solution provided only partial support for the proposed structure. Absolute primary loadings ranged from 0.296 to 0.769, maximum absolute cross-loadings ranged from 0.057 to 0.365, and communalities ranged from 0.328 to 0.640. Twenty-four of the 40 items met all four prespecified interpretive criteria: a primary loading ≥ 0.40, maximum cross-loading < 0.30, primary-to-cross-loading difference ≥ 0.20, and communality ≥ 0.30. The complete rotated pattern matrix is reported in Figure 1 and the accompanying item-level results.

**Figure 1:**
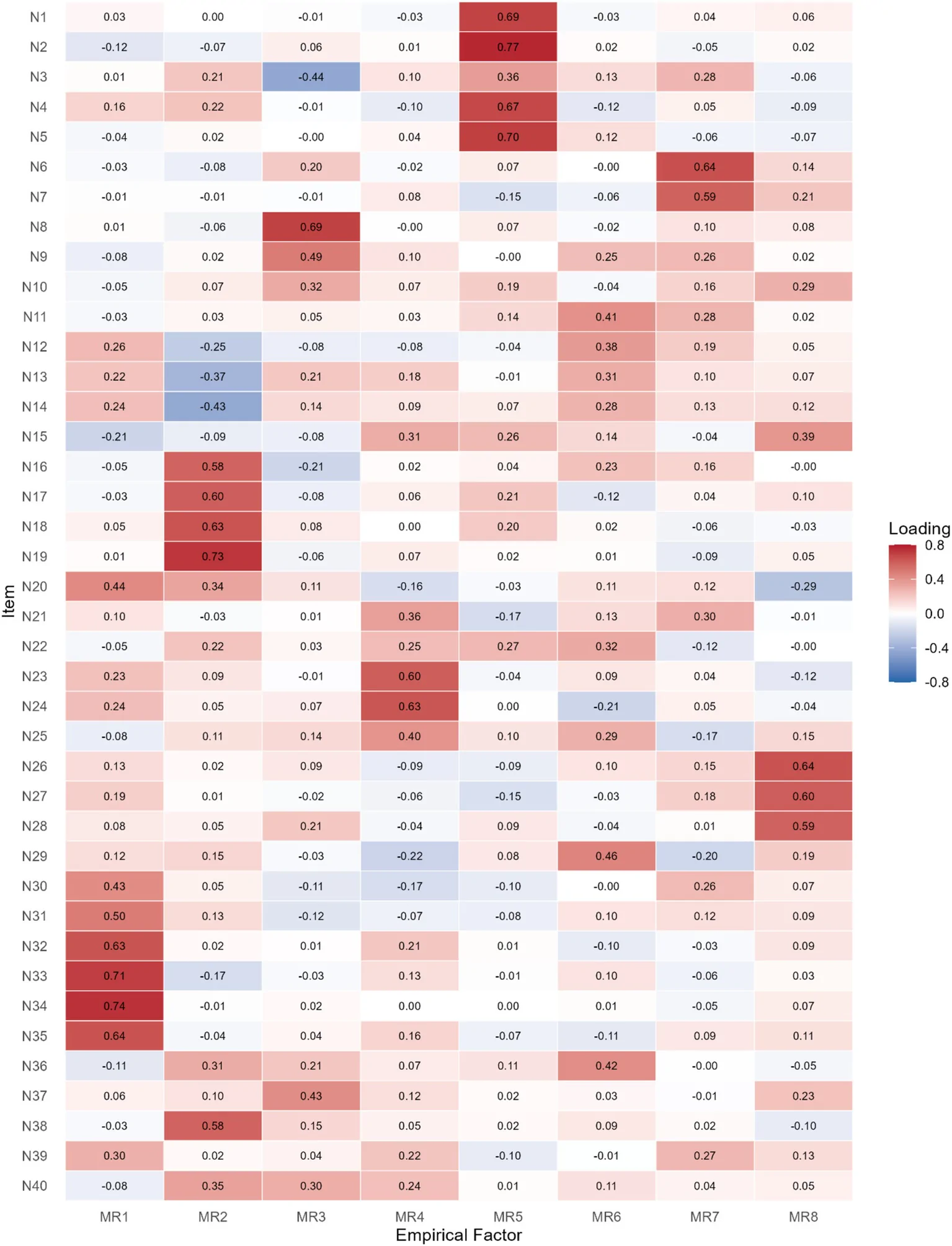
Eight-factor EFA pattern matrix in the 1,052-participant sample.

The proposed correlated eight-factor model was subsequently examined in an independent sample of 1,022 participants who were not included in the EFA sample. Confirmatory factor analysis was performed using weighted least squares mean- and variance-adjusted estimation, with the items treated as ordered categorical variables. The model converged but showed inadequate fit: scaled χ^2^(712) = 11011.37, *p* < 0.001, CFI = 0.691, TLI = 0.661, RMSEA = 0.119 (90% CI [0.117, 0.121]), and SRMR = 0.123. Thus, the independent CFA did not confirm the proposed eight-factor structure. The eight-domain specification was retained as the theory-based scoring framework used in the present network analysis, but its factorial validity should be regarded as provisional.

The final Q8PS is a 40-item self-report measure assessing Yanggang, Yinrou, Introvertion, Extraversion, Sensitivity, Deliberation, Contradiction, and Harmoniousness. Items are rated on a 6-point Likert scale from 1 (strongly disagree) to 6 (strongly agree). Each subscale contains five items that are summed, with higher scores indicating a stronger expression of the corresponding trait.

In the present 1,052-participant sample, Cronbach’s α was 0.839 for the total Q8PS and ranged from 0.58 to 0.81 across the eight subscales (Table 4). Together with the mixed factor-analytic findings, these reliability estimates indicate that the psychometric evidence for the current 40-item scale remains provisional.

**Table 4:** Distribution of dimension.

Variables	*M*	SD	Median	Max	Min	Sk	Kr	Cronbach’s α	Mean corrected item–total correlation
Q8P Yanggang	17.16	4.8	17	30	5	0.1	−0.28	0.78	0.56
Q8P Yinrou	15.2	4.72	15	29	5	0.15	−0.31	0.71	0.47
Q8P introvertion	15.92	4.49	16	30	5	0	−0.24	0.63	0.38
Q8P extraversion	15.84	4.67	16	30	5	0.31	0.04	0.75	0.52
Q8P sensitivity	17.65	4.25	18	30	5	0.11	−0.03	0.64	0.4
Q8P deliberation	14.99	4.24	15	30	5	0.21	−0.17	0.63	0.39
Q8P contradiction	16.2	5.32	16	30	5	0.09	−0.4	0.81	0.6
Q8P harmoniousness	16.84	3.94	17	30	5	0.04	0.33	0.58	0.34
BFP neuroticism	33.96	4.96	34	53	17	−0.11	0.29	0.829	0.484
BFP extraversion	37.76	5.19	38	58	18	−0.04	0.63	0.720	0.357
BFP openness	36.57	4.77	37	58	18	0.24	1.73	0.362	0.132
BFP agreeableness	34	4.94	34	54	14	0.13	0.64	0.443	0.173
BFP conscientiousness	39.44	5.02	40	59	17	−0.45	0.98	0.707	0.359

Study variables: means, standard deviations, skewness, and kurtosis, Cronbach’s alpha *n* = 1,052.

#### 2.2.2 Chinese Big Five inventory–2 (BFI-2)

Personality traits were assessed using the Chinese BFI-2 (Zhang et al., 2022). The instrument contains 60 self-report items measuring neuroticism, extraversion, openness, agreeableness, and conscientiousness, with 12 items per domain. Items are rated from 1 (strongly disagree) to 5 (strongly agree). The 24 reverse-keyed items were recoded as 6 minus the observed response before the 12 items in each domain were summed. In this study, the BFI-2 was used as an established comparison framework, not as an error-free gold standard or as sufficient evidence by itself for validating the Q8PS.

The psychometric performance of the BFI-2 was evaluated in the present 1,052-participant sample using all 60 items. A correlated five-factor CFA was estimated with robust maximum likelihood (MLR) in lavaan version 0.6–19, with WLSMV for ordered items as a sensitivity analysis. The conventional MLR indices were χ^2^/df = 4.967, RMSEA = 0.061 (90% CI [0.060, 0.063]), CFI = 0.578, TLI = 0.561, and SRMR = 0.086. The WLSMV analysis led to the same substantive conclusion (CFI = 0.663, TLI = 0.649, RMSEA = 0.073, and SRMR = 0.094). Raw-score Cronbach’s α values were 0.829 for neuroticism, 0.720 for extraversion, 0.362 for openness, 0.443 for agreeableness, and 0.707 for conscientiousness. Thus, the five-factor structure received limited rather than confirmatory support, and the low reliability of openness and agreeableness was considered when interpreting the network associations.

### 2.3 Statistical analysis

#### 2.3.1 Descriptive statistic

The sample characteristics were examined using descriptive statistics via R version 4.2.1.

#### 2.3.2 Network analysis

Following an initial assessment of the descriptive statistics (mean, standard deviation, kurtosis, and skewness) and internal consistency (Cronbach’s alpha) of the Q8PS and BFI-2 dimensions, we constructed a cross-sectional network comprising eight Q8PS dimensions and five BFI-2 domains. Each Q8PS dimension score was calculated by summing its five items, whereas each BFI-2 domain score was calculated by summing its 12 keyed items after the reverse-keyed items had been recoded. The analysis followed established reporting standards for cross-sectional psychological network studies (Burger et al., 2023).

Network analysis estimates and visualizes the conditional relationships among psychological variables (Epskamp et al., 2012, 2018). Compared with conventional correlation analyses, it allows the association between two nodes to be examined while controlling for all other nodes in the network (Bringmann and Eronen, 2018). This approach has previously been used to investigate relationships between dimensions derived from different personality questionnaires (Di Fabio et al., 2023; Zhang et al., 2025).

The network analysis was conducted as follows. First, zero-order associations among the 13 dimensions were calculated using Spearman rank correlations. The regularized partial correlation network was then estimated from a Pearson product–moment correlation matrix using the graphical least absolute shrinkage and selection operator with Extended Bayesian Information Criterion model selection (EBICglasso). The network was estimated using bootnet::estimateNetwork with default = “EBICglasso” and corMethod = “cor.” The EBIC hyperparameter (γ) was set to 0.50, and 100 candidate regularization values were evaluated with a minimum λ ratio of 0.01. The model was estimated without refitting or additional thresholding. Pairwise-complete observations and the pairwise-average sample-size rule were specified; however, no values were missing across the 13 variables in the final sample of 1,052 participants.

Second, we examined the nodes and edges, together with expected influence (EI) and bridge expected influence (BEI). The network comprised 13 nodes, of which eight represented Q8PS dimensions and five represented BFI-2 domains. Edges represented regularized partial correlations between two dimensions after controlling for the other 11 dimensions. Positive and negative edges indicated conditional associations in the corresponding directions, and edge thickness represented the absolute edge weight. A spring layout was used to visualize the weight matrix, but the spatial positions of the nodes were not interpreted inferentially.

EI was calculated as the signed sum of all edge weights connected to a node. Unlike strength centrality, EI retains the direction of negative associations and is therefore suitable for networks containing both positive and negative edges (Robinaugh et al., 2016). Higher EI values indicated that a node had relatively stronger overall connections with the other nodes in the network.

One-step BEI was calculated as the signed sum of the edges connecting a node to nodes in the other community. The two communities were defined *a priori* as the eight Q8PS dimensions and the five BFI-2 domains, respectively, and BEI was calculated using networktools::bridge. No numerical cutoff or statistical threshold was used to classify nodes categorically as bridge nodes. Therefore, BEI was interpreted as a continuous measure, and nodes were described as having relatively high or low BEI based on their estimates, rankings, uncertainty intervals, and bootstrap comparisons (Jones et al., 2021).

Third, we assessed the stability of EI and BEI using 1,000 case-dropping bootstrap samples. Between 5 and 75% of participants were progressively removed across 10 sampling levels. Correlation-stability (CS) coefficients were calculated using a correlation threshold of 0.70. The CS coefficient represents the maximum proportion of cases that can be removed while retaining, with 95% probability, a correlation of at least 0.70 between the centrality estimates obtained from the reduced and original samples. CS coefficients above 0.25 were considered acceptable, whereas values above 0.50 were considered good (Epskamp et al., 2018).

Finally, edge-weight accuracy was evaluated using 1,000 nonparametric bootstrap samples. For each edge, a 95% percentile bootstrap confidence interval was calculated from the 2.5th and 97.5th percentiles of its bootstrap distribution. Narrower confidence intervals indicated greater estimation precision, whereas wider intervals indicated greater uncertainty (Epskamp et al., 2018). Nonparametric bootstrap difference tests were also used to compare edge weights with one another and to compare EI and BEI values across nodes. These tests evaluated whether the bootstrap distribution of the difference between two estimates included zero; they did not test whether an individual edge was statistically different from zero. No multiplicity correction was applied because these comparisons were exploratory, and the difference-test results were interpreted cautiously rather than as confirmatory hypothesis tests.

All network analyses were performed using R version 4.4.1. Zero-order correlations were calculated using Hmisc::rcorr in Hmisc version 5.2–1. Network estimation, bootstrap analyses, and CS coefficients were obtained using bootnet::estimateNetwork, bootnet::bootnet, and bootnet::corStability in bootnet version 1.6. Network visualization was performed using qgraph::qgraph in qgraph version 1.9.8, and BEI was calculated using networktools::bridge in networktools version 1.6.0. A fixed random seed of 20,250,823 was used to ensure reproducibility.

## 3 Results

### 3.1 Descriptive statistic

Table 4 presents the descriptive statistics of the study variables, and Figure 2 displays the zero-order Spearman correlations among the 13 dimensions.

**Figure 2:**
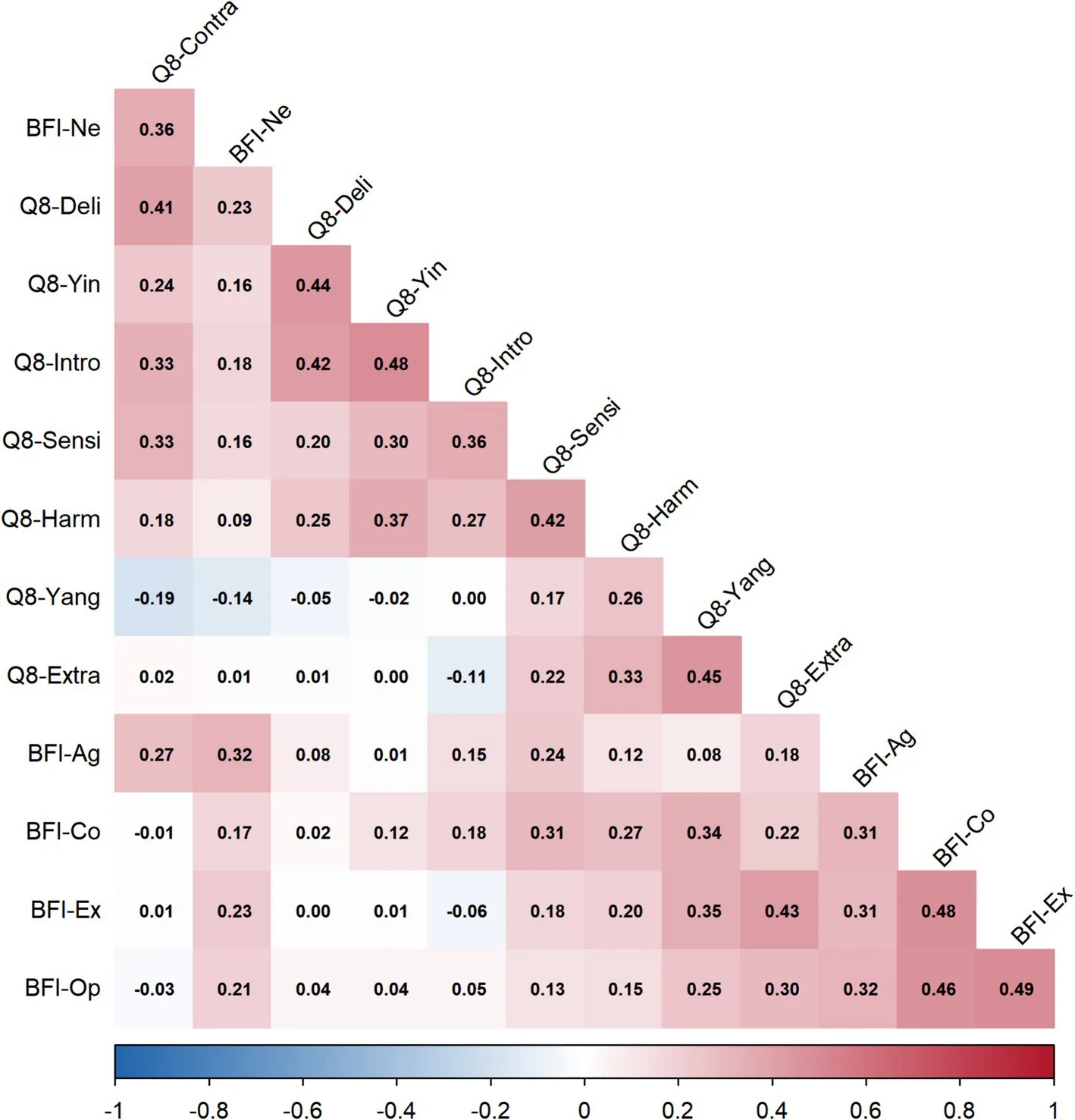
Zero-order Spearman correlations among the Q8PS and BFI-2 dimensions (*n* = 1,052). Q8-Yang, Yanggang; Q8-Yin, Yinrou; Q8-Intro, Introvertion; Q8-Extra, Extraversion; Q8-Sensi, Sensitivity; Q8-Deli, Deliberation; Q8-Contra, Contradiction; Q8-Harm, Harmoniousness; BFP-Ne, neuroticism; BFP-Ex, extraversion; BFP-Op, openness(tentative); BFP-Ag, agreeableness(tentative); and BFP-Co, conscientiousness.

Cronbach’s α for the complete 40-item Q8PS was 0.839, and the eight Q8PS subscale coefficients ranged from 0.58 to 0.81. For the BFI-2 domains, α was 0.829 for neuroticism, 0.720 for extraversion, 0.362 for openness, 0.443 for agreeableness, and 0.707 for conscientiousness. The low openness and agreeableness coefficients indicate substantial measurement uncertainty for network associations involving these domains.

### 3.2 Network analysis

#### 3.2.1 Network model

Figure 3 illustrates the EBICglasso regularized partial correlation network linking the Q8PS and BFI-2 dimensions. Node colors indicate the two prespecified communities. Blue edges represent positive conditional associations, red edges represent negative conditional associations, and thicker edges indicate larger absolute edge weights.

**Figure 3:**
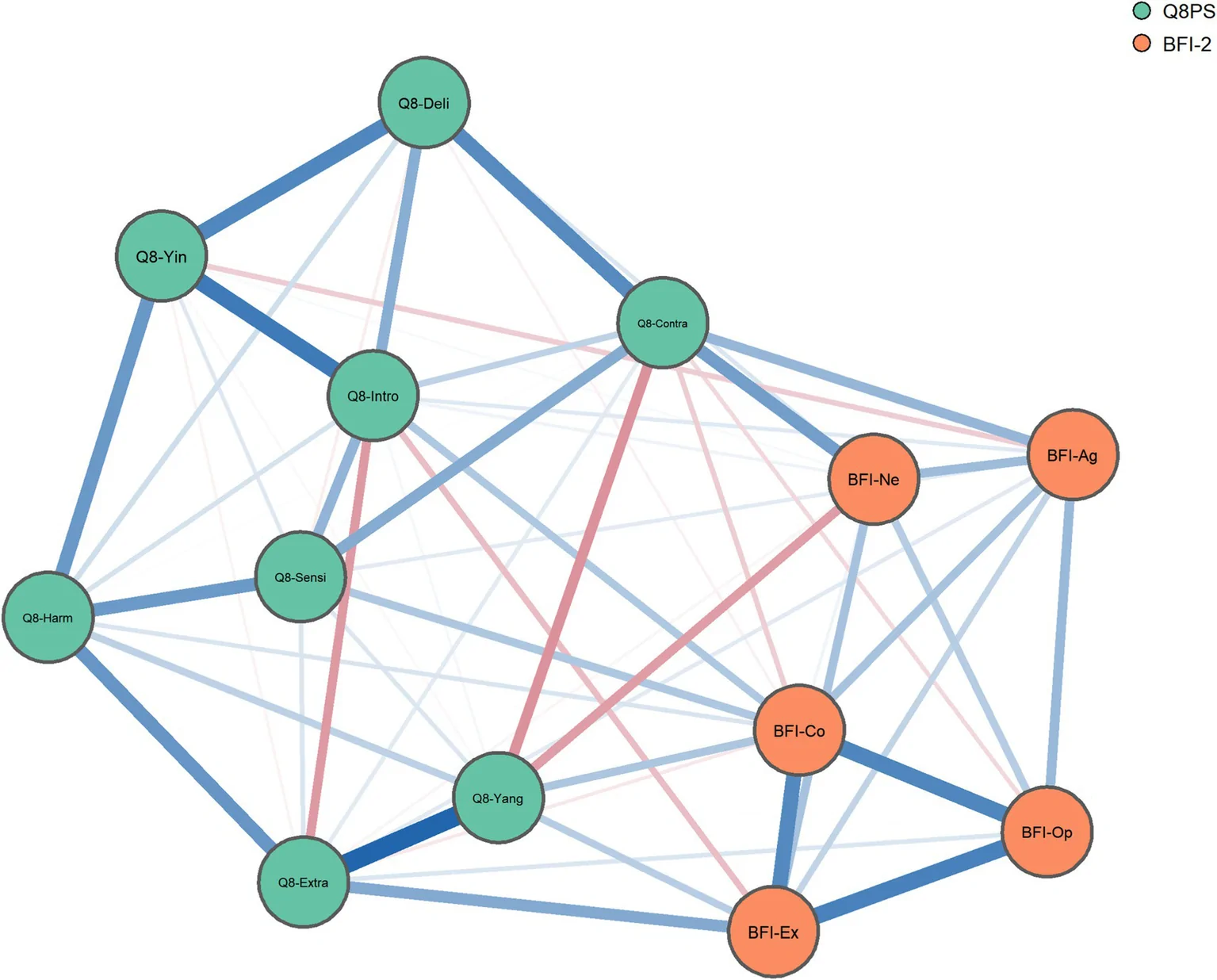
EBICglasso regularized partial correlation network of the Q8PS and BFP dimensions (*n* = 1,052). Notes: Each node represents one Q8PS or BFP dimension. Blue and red edges represent positive and negative regularized partial correlations, respectively; thicker and more saturated edges indicate larger absolute weights. The spatial layout is provided for visualization and has no inferential meaning. Q8-Yang, Yanggang; Q8-Yin, Yinrou; Q8-Intro, Introvertion; Q8-Extra, Extraversion; Q8-Sensi, Sensitivity; Q8-Deli, Deliberation; Q8-Contra, Contradiction; Q8-Harm, Harmoniousness; BFP-Ne, neuroticism; BFP-Ex, extraversion; BFP-Op, openness(tentative); BFP-Ag, agreeableness(tentative); and BFP-Co, conscientiousness.

The network contained 78 possible edges, of which 60 were nonzero (76.92%): 47 were positive and 13 were negative. The estimated edge weights ranged from −0.160 to 0.339. Table 5 presents the complete regularized weight matrix.

**Table 5:** Weight matrix for the EBICglasso regularized partial correlation network.

Variables	1	2	3	4	5	6	7	8	9	10	11	12	13
1. Q8P Yanggang	—												
2. Q8P Yinrou	−0.01	—											
3. Q8P introvertion	0.02	0.30	—										
4. Q8P extraversion	0.34	−0.02	−0.15	—									
5. Q8P sensitivity	0.06	0.05	0.17	0.06	—								
6. Q8P deliberation	0.00	0.27	0.18	0.00	−0.03	—							
7. Q8P contradiction	−0.16	0.00	0.10	0.05	0.19	0.26	—						
8. Q8P harmoniousness	0.10	0.23	0.06	0.23	0.22	0.07	0.01	—					
9. BFP neuroticism	−0.14	0.01	0.03	−0.02	0.00	0.06	0.21	0.00	—				
10. BFP extraversion	0.11	0.00	−0.10	0.19	0.00	0.00	0.00	0.00	0.13	—			
11. BFP openness(tentative)	0.00	0.00	0.00	0.06	0.00	0.00	−0.06	0.00	0.11	0.28	—		
12. BFP agreeableness(tentative)	0.00	−0.07	0.05	0.05	0.04	0.00	0.16	0.00	0.15	0.10	0.16	—	
13. BFP conscientiousness	0.13	0.00	0.12	−0.03	0.12	−0.02	−0.07	0.06	0.02	0.26	0.27	0.13	—

The largest cross-framework edge connected Q8P Contradiction and BFP neuroticism (weight = 0.215), followed by Q8P Extraversion and BFP extraversion (weight = 0.190).

Q8P Contradiction was also positively connected with BFP agreeableness (weight = 0.161) (tentative). These three positive edges were the most prominent cross-framework associations in the estimated network.

The largest negative cross-framework edge connected Q8P Yanggang and BFP neuroticism (weight = −0.142).

BFP conscientiousness was positively connected with Q8P Yanggang (weight = 0.126), Sensitivity (weight = 0.124), and Introvertion (weight = 0.122).

Additional cross-framework edges were smaller in absolute magnitude, including Q8P Yanggang–BFP extraversion (weight = 0.110) and Q8P Introvertion–BFP extraversion (weight = −0.097).

The remaining cross-framework edge weights were below 0.10 in absolute value.

Although these edges were the largest cross-framework connections relative to the other estimated edges, their absolute magnitudes were small to moderate.

Accordingly, the network findings were interpreted in terms of relative prominence and estimation uncertainty rather than as evidence of large effects.

#### 3.2.2 Expected influence and bridge expected influence

The EI and BEI estimates, their ranks, and 95% bootstrap confidence intervals are presented in Figures 4–6 and Table 6. Q8P Harmoniousness (EI = 0.993, 95% CI [0.867, 1.103]), BFI-2 conscientiousness (EI = 0.993, 95% CI [0.886, 1.088]), and BFI-2 extraversion (EI = 0.976, 95% CI [0.865, 1.081]) showed the highest EI estimates. Q8P Yanggang showed the lowest EI (EI = 0.438, 95% CI [0.334, 0.548]). These rankings were interpreted relatively because several intervals overlapped.

**Figure 4:**
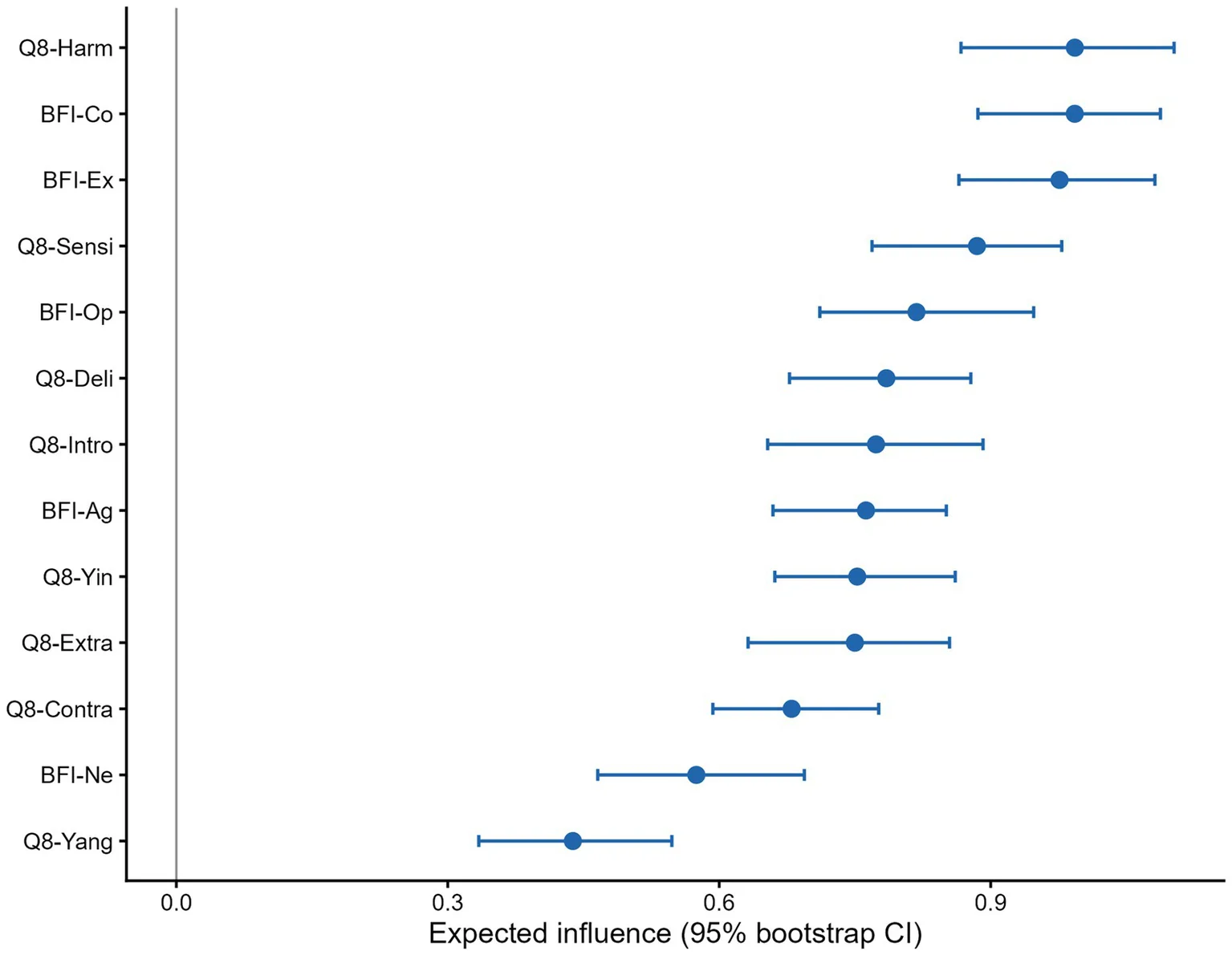
Expected influence estimates with 95% nonparametric bootstrap confidence intervals (*n* = 1,052). Points represent EI estimates and horizontal bars represent 95% percentile bootstrap confidence intervals based on 1,000 resamples. Q8-Yang, Yanggang; Q8-Yin, Yinrou; Q8-Intro, Introvertion; Q8-Extra, Extraversion; Q8-Sensi, Sensitivity; Q8-Deli, Deliberation; Q8-Contra, Contradiction; Q8-Harm, Harmoniousness; BFP-Ne, neuroticism; BFP-Ex, extraversion; BFP-Op, openness(tentative); BFP-Ag, agreeableness(tentative); and BFP-Co, conscientiousness.

**Figure 5:**
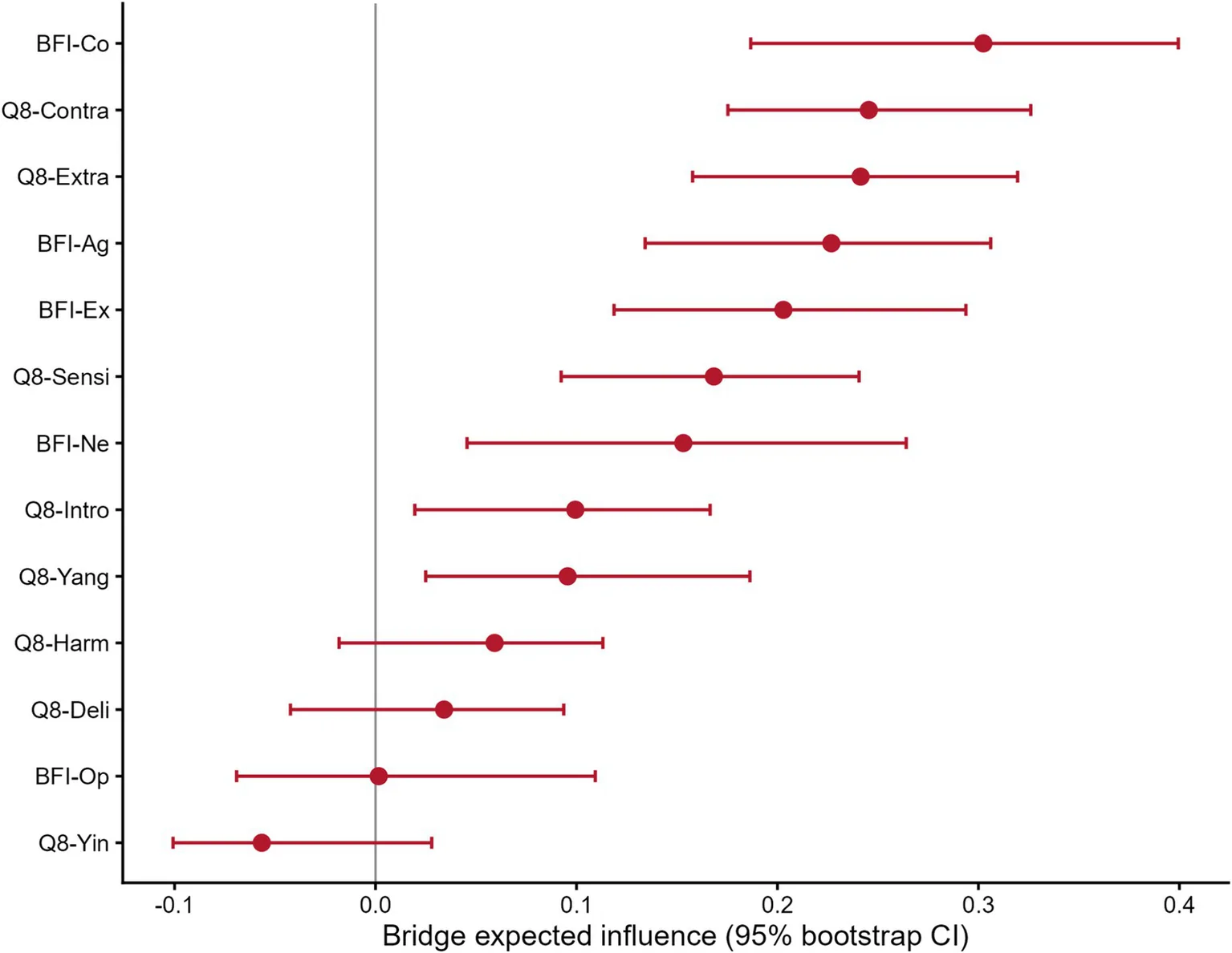
Bridge expected influence estimates with 95% nonparametric bootstrap confidence intervals (*n* = 1,052). Points represent one-step BEI estimates and horizontal bars represent 95% percentile bootstrap confidence intervals based on 1,000 resamples. Q8-Yang, Yanggang; Q8-Yin, Yinrou; Q8-Intro, Introvertion; Q8-Extra, Extraversion; Q8-Sensi, Sensitivity; Q8-Deli, Deliberation; Q8-Contra, Contradiction; Q8-Harm, Harmoniousness; BFP-Ne, neuroticism; BFP-Ex, extraversion; BFP-Op, openness(tentative); BFP-Ag, agreeableness(tentative); and BFP-Co, conscientiousness.

**Figure 6:**
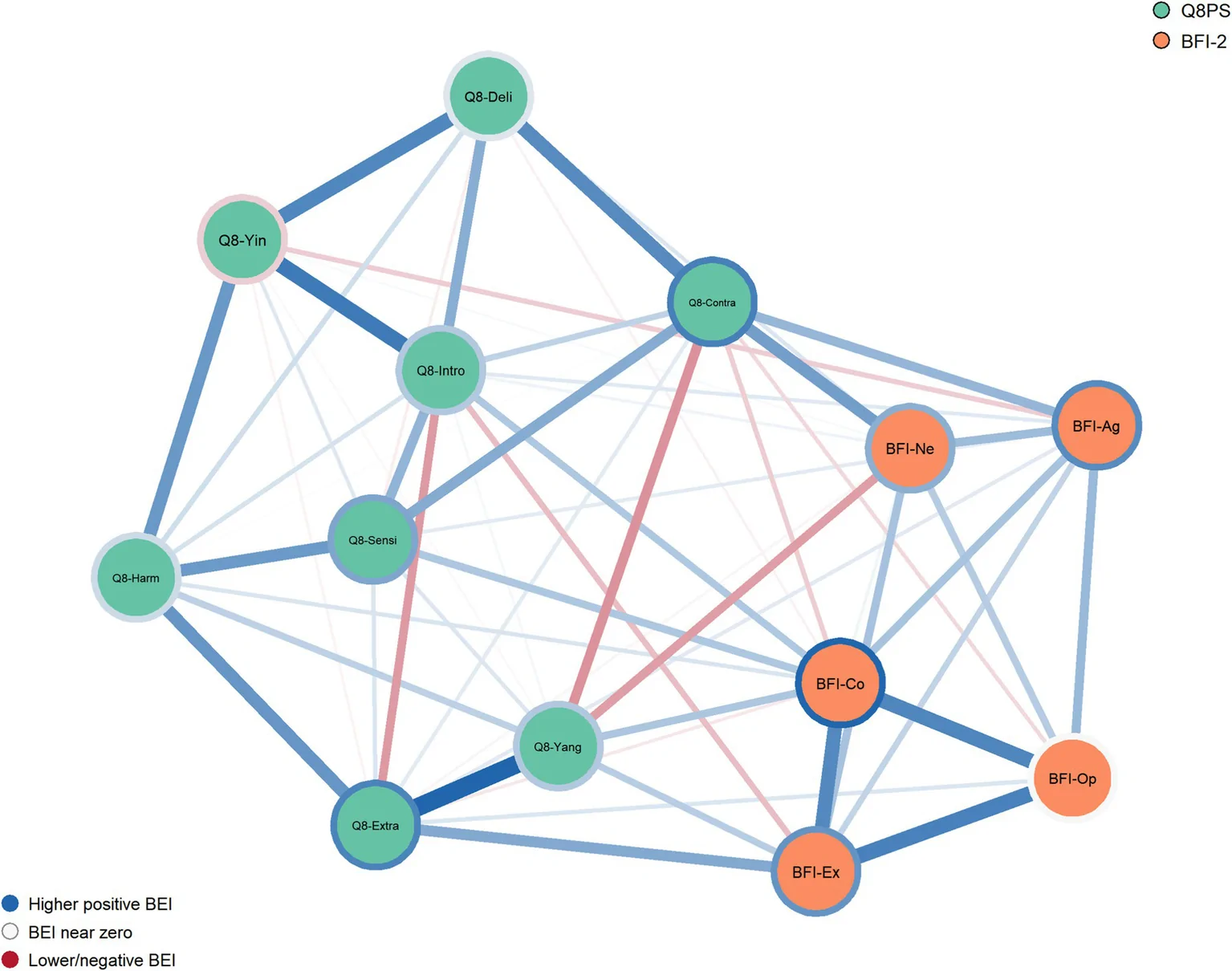
Node-border color maps one-step bridge expected influence (BEI) continuously on a diverging red -white -blue scale: red indicates lower or negative BEI values, white indicates values near zero, and blue indicates higher positive BEI values. These colors represent positions on a continuous scale and do not classify nodes into bridge and non-bridge categories. Blue and red edges indicate positive and negative regularized partial correlations, respectively; thicker and more saturated edges indicate larger absolute edge weights. Node-border colors represent point estimates only; the corresponding 95% bootstrap confidence intervals are reported in Figure 5 and Table 6. Q8-Yang, Yanggang; Q8-Yin, Yinrou; Q8-Intro, Introvertion; Q8-Extra, Extraversion; Q8-Sensi, Sensitivity; Q8-Deli, Deliberation; Q8-Contra, Contradiction; Q8-Harm, Harmoniousness; BFI-Ne, neuroticism; BFI-Ex, extraversion; BFI-Op, openness(tentative); BFI-Ag, agreeableness(tentative); and BFI-Co, conscientiousness.

**Table 6:** Expected influence and bridge expected influence estimates, ranks, and 95% bootstrap confidence intervals.

Variables	Community	EI (rank)	EI 95% CI	BEI (rank)	BEI 95% CI
Q8P Yanggang	Q8PS	0.438 (13)	[0.334, 0.548]	0.096 (9)	[0.025, 0.186]
Q8P Yinrou	Q8PS	0.753 (9)	[0.661, 0.861]	−0.057 (13)	[−0.101, 0.028]
Q8P introvertion	Q8PS	0.773 (7)	[0.653, 0.892]	0.099 (8)	[0.020, 0.166]
Q8P extraversion	Q8PS	0.750 (10)	[0.632, 0.855]	0.241 (3)	[0.158, 0.319]
Q8P sensitivity	Q8PS	0.885 (4)	[0.769, 0.979]	0.168 (6)	[0.092, 0.241]
Q8P deliberation	Q8PS	0.785 (6)	[0.678, 0.878]	0.034 (11)	[−0.042, 0.094]
Q8P contradiction	Q8PS	0.680 (11)	[0.593, 0.776]	0.245 (2)	[0.175, 0.326]
Q8P harmoniousness	Q8PS	0.993 (1)	[0.867, 1.103]	0.059 (10)	[−0.018, 0.113]
BFP neuroticism	BFP	0.575 (12)	[0.466, 0.694]	0.153 (7)	[0.046, 0.264]
BFP extraversion	BFP	0.976 (3)	[0.865, 1.081]	0.203 (5)	[0.119, 0.294]
BFP openness(tentative)	BFP	0.818 (5)	[0.711, 0.948]	0.002 (12)	[−0.069, 0.109]
BFP agreeableness(tentative)	BFP	0.762 (8)	[0.659, 0.851]	0.227 (4)	[0.134, 0.306]
BFP conscientiousness	BFP	0.993 (2)	[0.886, 1.088]	0.302 (1)	[0.187, 0.399]

EI and one-step BEI are reported as unstandardized signed sums of edge weights. Ranks are shown in parentheses, with 1 indicating the highest estimate. Confidence intervals are 95% percentile intervals from 1,000 nonparametric bootstrap samples. Figures 5, 6 present BEI as a continuous measure. Figure 5 shows the node-specific estimates and uncertainty intervals, whereas Figure 6 displays the same BEI information continuously through node-border color within the estimated network.

Under the prespecified two-community definition, BFP conscientiousness had the highest BEI (BEI = 0.302, 95% CI [0.187, 0.399]), followed by Q8P Contradiction (BEI = 0.245, 95% CI [0.175, 0.326]), Q8P Extraversion (BEI = 0.241, 95% CI [0.158, 0.319]), BFP agreeableness (BEI = 0.227, 95% CI [0.134, 0.306]) (tentative), and BFP extraversion (BEI = 0.203, 95% CI [0.119, 0.294]). Bootstrap difference tests did not reliably distinguish BFP conscientiousness from these other relatively high-BEI nodes. Q8P Harmoniousness ranked tenth (BEI = 0.059, 95% CI [−0.018, 0.113]). Therefore, no nodes were classified categorically as statistically identified bridge nodes.

#### 3.2.3 Bootstrap test results

Figure 7 presents the nonparametric bootstrap difference matrix for the nonzero edge weights. Black cells indicate pairs of edges whose bootstrap difference interval excluded zero, whereas grey cells indicate pairs that were not reliably distinguishable. These comparisons concern differences between edge weights and do not establish that an individual edge is statistically significant.

**Figure 7:**
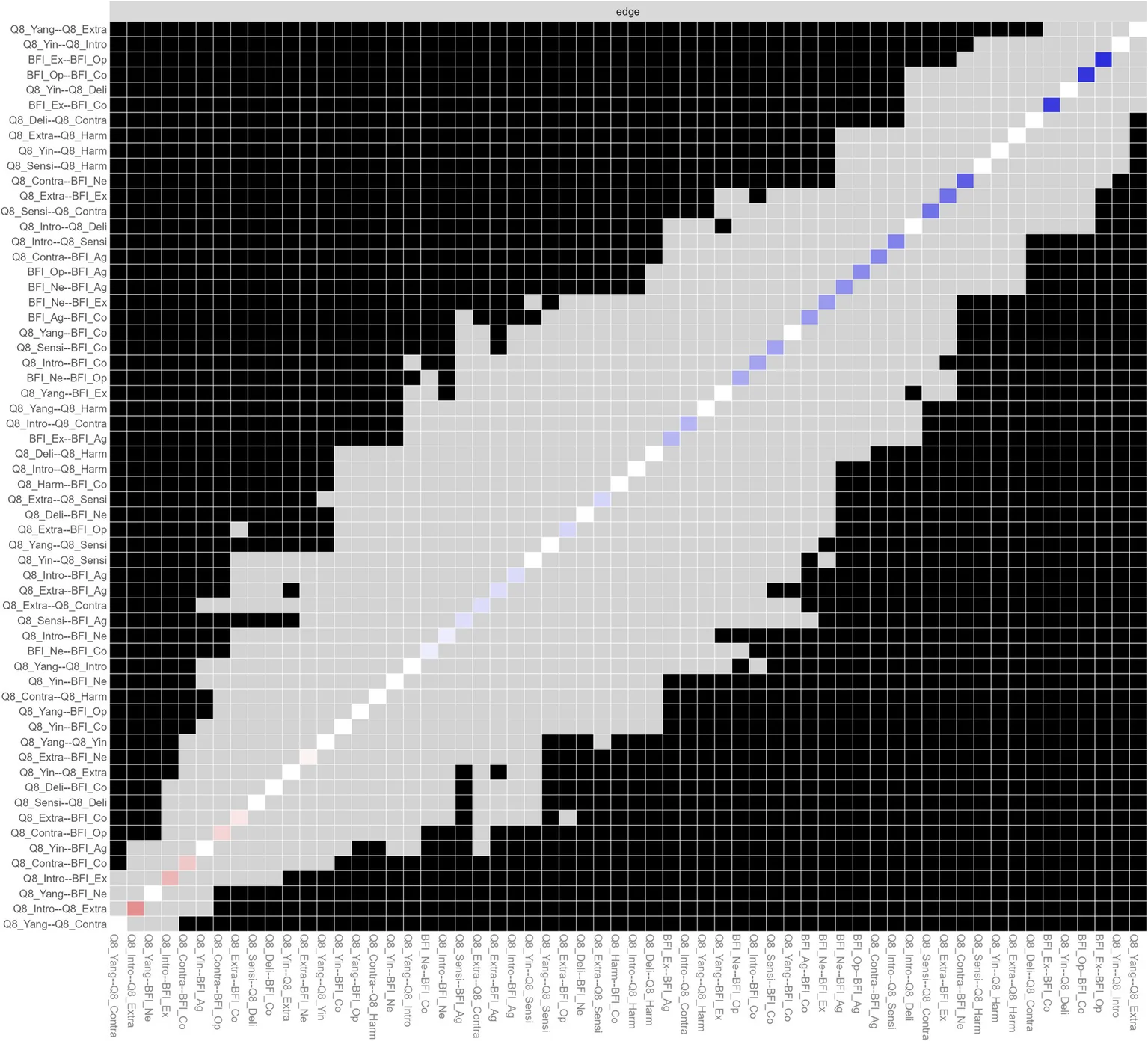
Nonparametric bootstrap difference test comparing nonzero edge weights (1,000 resamples; *n* = 1,052). The axes list the nonzero edge estimates from lowest to highest. Black cells indicate that the bootstrap interval for the difference between two edge weights excluded zero; gray cells indicate that it included zero. The diagonal shows the magnitude and direction of each edge. This figure compares edge weights with one another and does not test the significance of an individual edge.

Figure 8 presents the accuracy analysis for all edge weights. The principal cross-framework estimates and 95% bootstrap confidence intervals were: Q8P Contradiction–BFP neuroticism, 0.215 [0.157, 0.268]; Q8P Extraversion–BFP extraversion, 0.190 [0.133, 0.248]; Q8P Contradiction–BFPagreeableness, 0.161 [0.103, 0.224]; and Q8P Yanggang–BFP neuroticism, −0.142 [−0.200, −0.075]. The intervals quantify estimation uncertainty; they should not be interpreted solely from the visual prominence of an edge.

**Figure 8:**
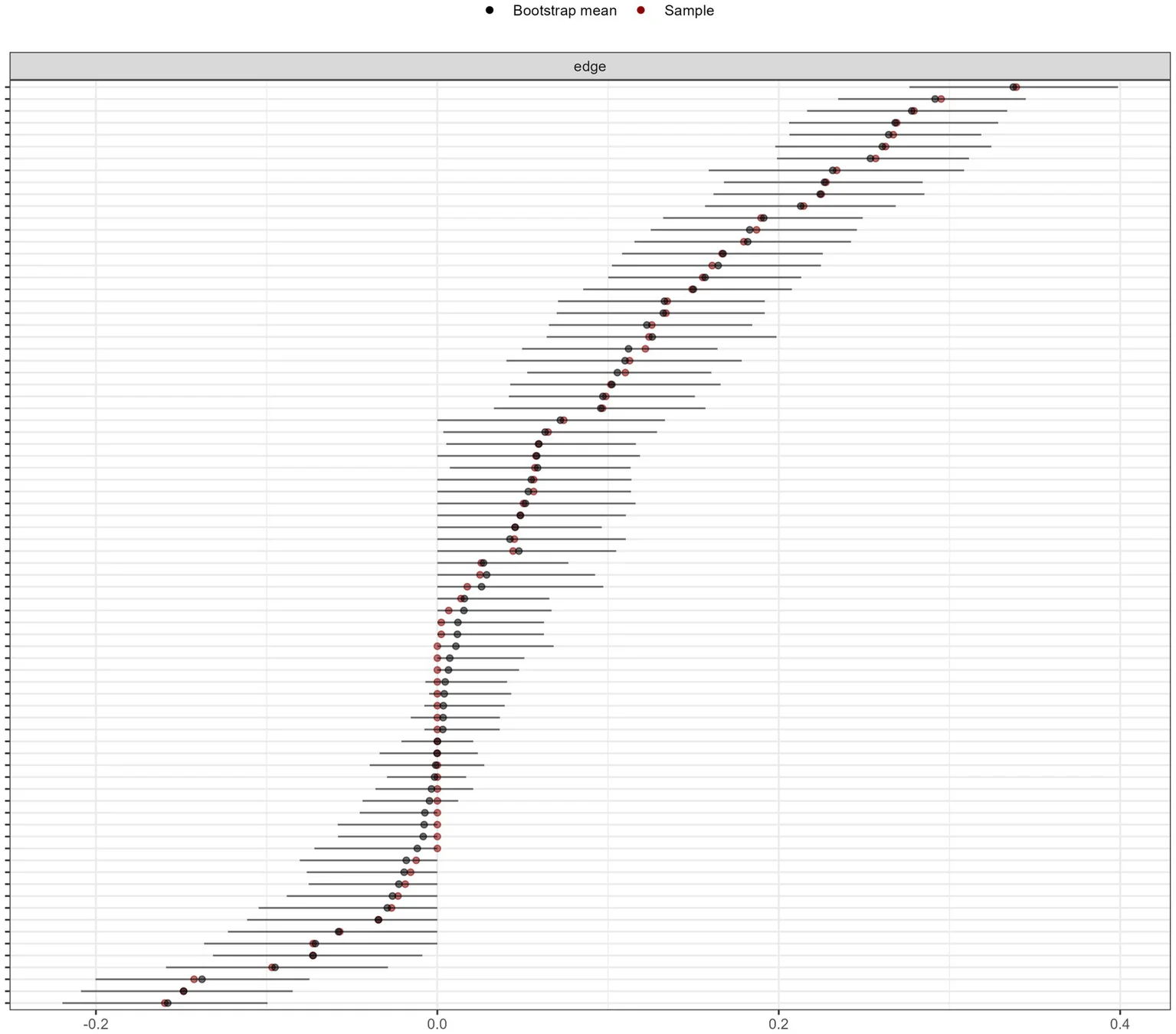
Accuracy of edge-weight estimates based on 1,000 nonparametric bootstrap samples (*n* = 1,052). Horizontal lines represent 95% percentile bootstrap confidence intervals, black points represent bootstrap means, and red points represent the estimates from the original sample. Edges are ordered by their original-sample weights.

Figures 9, 10 present bootstrap difference tests for EI and BEI, respectively. The separate case-dropping analysis shown in Figure 11 produced CS coefficients of 0.672 for EI and 0.750 for BEI, indicating good stability for both measures. A CS coefficient was not used to assess individual edges because edge accuracy was evaluated using the nonparametric bootstrap confidence intervals in Figure 8.

**Figure 9:**
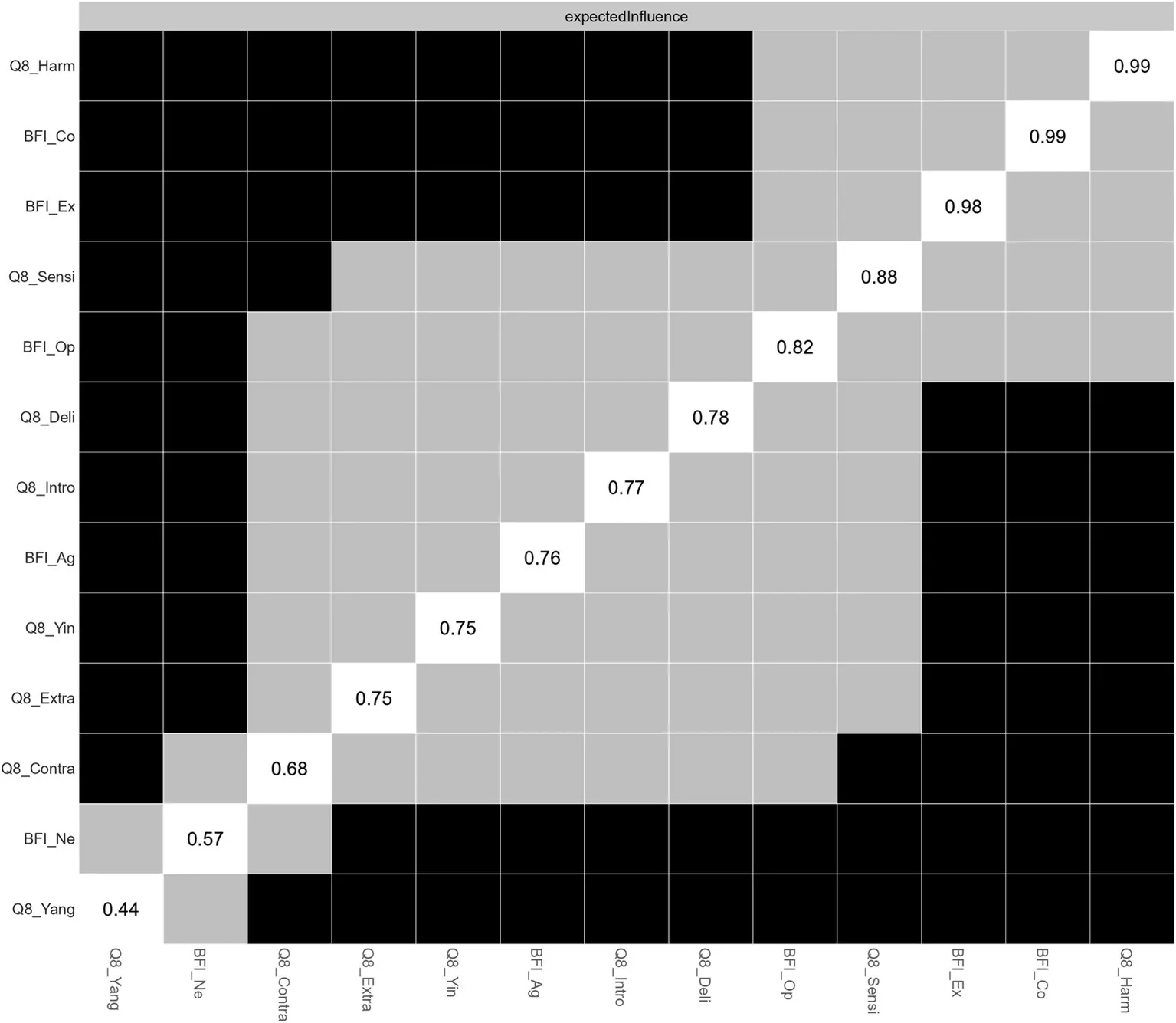
Nonparametric bootstrap difference test for expected influence (1,000 resamples; *n* = 1,052). The axes list EI estimates from lowest to highest. Black cells indicate that the bootstrap interval for the difference between two EI estimates excluded zero; grey cells indicate that it included zero. Q8-Yang, Yanggang; Q8-Yin, Yinrou; Q8-Intro, Introvertion; Q8-Extra, Extraversion; Q8-Sensi, Sensitivity; Q8-Deli, Deliberation; Q8-Contra, Contradiction; Q8-Harm, Harmoniousness; BFP-Ne, neuroticism; BFP-Ex, extraversion; BFP-Op, openness(tentative); BFP-Ag, agreeableness(tentative); and BFP-Co, conscientiousness.

**Figure 10:**
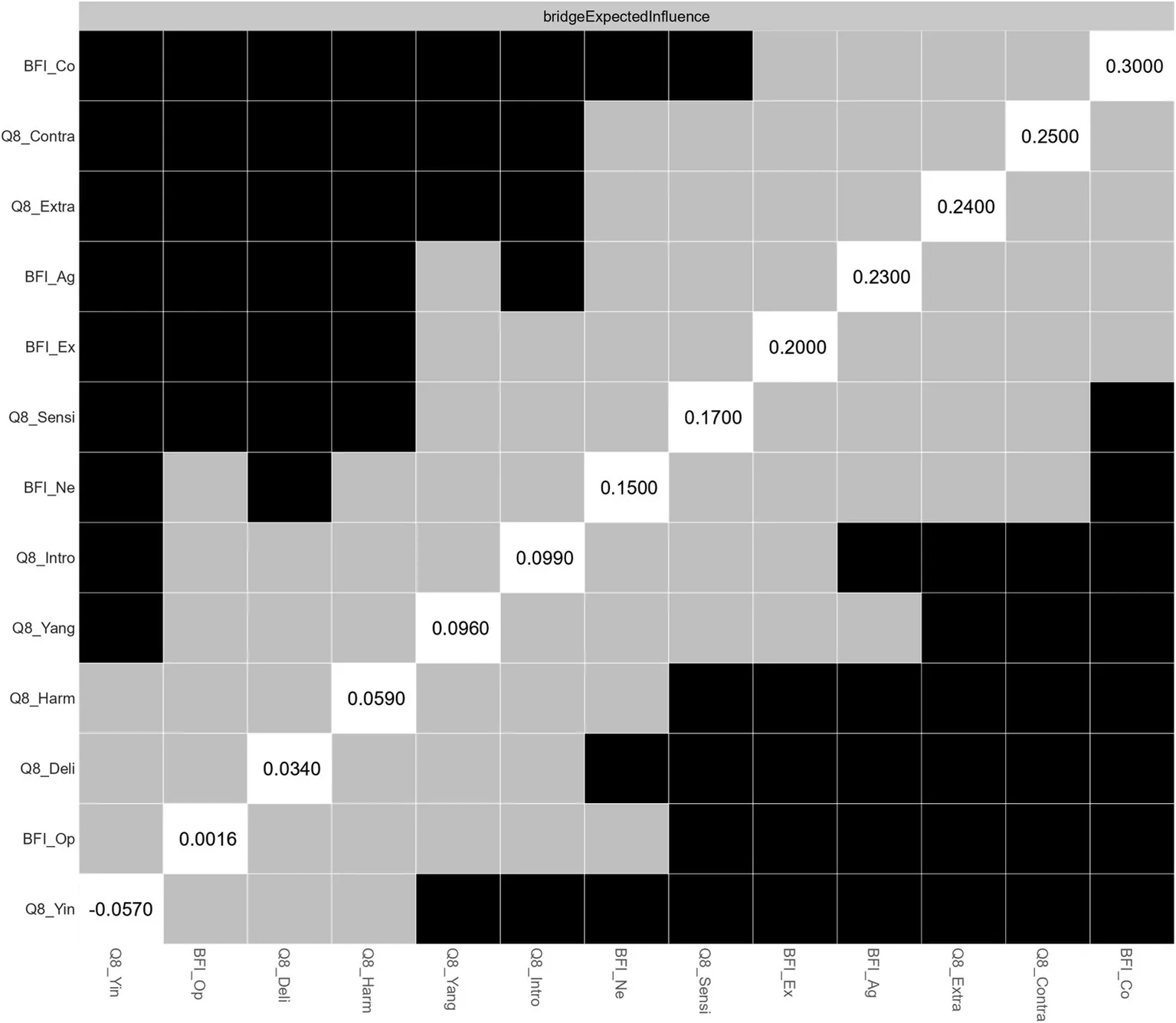
Nonparametric bootstrap difference test for bridge expected influence (1,000 resamples; *n* = 1,052). The axes list one-step BEI estimates from lowest to highest. Black cells indicate that the bootstrap interval for the difference between two BEI estimates excluded zero; grey cells indicate that it included zero. Q8-Yang, Yanggang; Q8-Yin, Yinrou; Q8-Intro, Introvertion; Q8-Extra, Extraversion; Q8-Sensi, Sensitivity; Q8-Deli, Deliberation; Q8-Contra, Contradiction; Q8-Harm, Harmoniousness; BFP-Ne, neuroticism; BFP-Ex, extraversion; BFP-Op, openness(tentative); BFP-Ag, agreeableness(tentative); and BFP-Co, conscientiousness.

**Figure 11:**
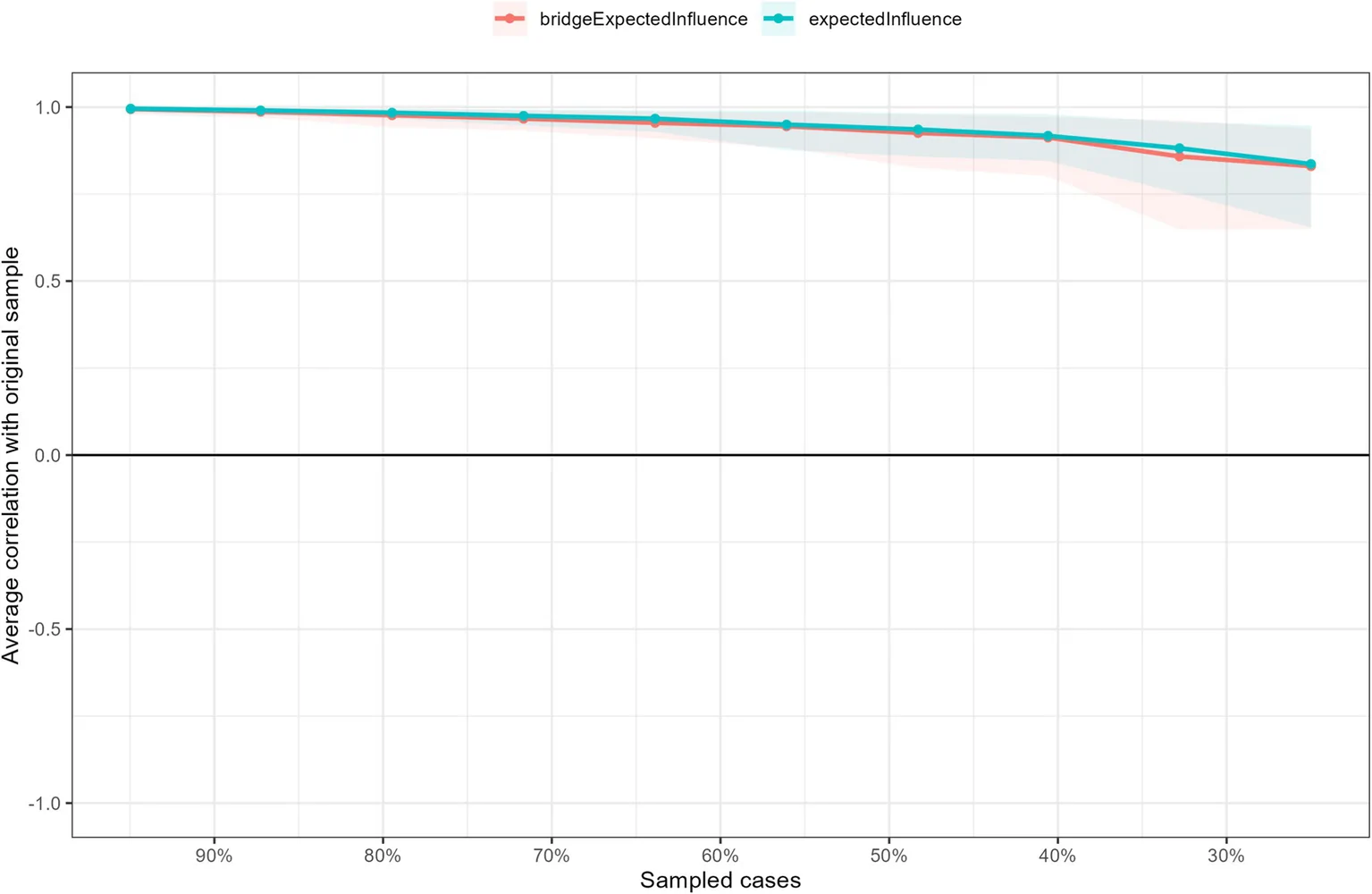
Case-dropping bootstrap stability of expected influence and bridge expected influence (1,000 resamples; *n* = 1,052). Lines show the average correlations between EI or BEI estimates obtained from case-dropping subsets and the corresponding estimates from the original sample; shaded areas represent 95% intervals. The CS coefficient was 0.672 for EI and 0.750 for BEI using a correlation threshold of 0.70.

## 4 Discussion

This study examined conditional associations between eight Q8P dimensions and five BFP domains. The largest cross-framework edges connected Q8P Contradiction with BFP neuroticism, Q8P Extraversion with BFP extraversion, and Q8P Yanggang with BFP conscientiousness. Although these edges were relatively prominent within the estimated network, their absolute magnitudes were small to moderate and should not be interpreted as evidence that the paired constructs are equivalent.

The positive Q8P Contradiction–BFP neuroticism association is consistent with shared negative-emotional content, whereas the Q8P Extraversion–BFP extraversion and Q8P Yanggang–BFP conscientiousness associations indicate partial correspondence in sociability and goal-directed responsibility. These patterns are descriptive associations among summed domain scores. They do not, by themselves, establish convergent validity, because the present study did not estimate a multitrait–multimethod measurement model or compare the constructs with prespecified external validity criteria.

The remaining Q8P dimensions showed more diffuse profiles across the BFP domains rather than clear one-to-one correspondence. Such patterns may help generate hypotheses about culturally grounded personality content, but they do not demonstrate that these dimensions are unique to the Q8PS or provide incremental validity beyond the BFP. Establishing those claims would require direct comparisons with validated measures and prediction of theoretically relevant external outcomes.

We therefore removed the earlier speculative comparisons linking selected Q8P dimensions to HEXACO honesty–humility or emotional intelligence. Neither HEXACO nor a validated emotional-intelligence measure was administered in this study, so those proposed connections cannot be evaluated empirically with the present data.

Bridge expected influence was interpreted continuously rather than used to classify statistically significant bridge nodes. BFP conscientiousness had the highest estimated BEI, but bootstrap comparisons did not reliably distinguish it from several other relatively high-BEI nodes. Accordingly, the bridge results identify relative patterns of cross-framework connectivity rather than categorical bridge traits.

Taken together, the network provides an exploratory map of how scores from the two frameworks relate in this Chinese adult sample. Interpretation should remain limited by the Q8PS’s provisional factorial validity, the reliability of several subscales, the convenience-sampling design, and the cross-sectional nature of the data (Table 7).

**Table 7:** Definitions of key constructs.

Dimensions	Definition	Representative figures
Yanggang	A tendency toward persistence, resilience, responsibility, assertiveness, and leadership-oriented behavior.	Superman
Yinrou	A tendency toward receptivity, nurturance, interpersonal softness, and willingness to accommodate others.	Princess Diana
Introvertion	A tendency toward restraint, low outward expressiveness, limited social engagement, and preference for introspection.	Stephen Hawking
Extraversion	A tendency toward social expressiveness, impulsive engagement, and active public participation.	Lady Gaga
Sensitivity	A tendency toward rapid perceptual and affective responsiveness, attention to detail, and heightened emotional reactivity.	Sherlock Holmes (Arthur Conan Doyle’s stories)
Deliberation	A tendency toward a slow, cautious response style, preference for stable pacing, and careful rather than rapid action.	Forrest Gump (Forrest Gump)
Harmoniousness	A tendency toward interpersonal attunement, flexibility, accommodation, and maintenance of relational harmony; it is not treated as a direct measure of emotional intelligence.	*Fred Rogers (Mister Rogers’ Neighborhood)*
Contradiction	A tendency toward ambivalence, internal conflict, indecisiveness, and fluctuating negative emotion.	Hamlet (William Shakespeare’s play *Hamlet*)
BFP Openness	Reflects curiosity, imagination, open-mindedness, and receptivity to novel ideas, experiences, and non-traditional values.	
Conscientiousness	Describes individuals who are organized, responsible, dependable, goal-oriented, and self-disciplined.	
Extraversion	Characterized by outgoingness, high energy, sociability, assertiveness, and a tendency to experience positive emotions.	
Agreeableness	Encompasses compassion, cooperativeness, trust, and forgiveness in interpersonal contexts.	
Neuroticism	Involves a propensity to experience negative emotions such as anxiety, anger, and depression; indicates low emotional stability.	

## 5 Conclusion

This exploratory network study identified several modest conditional associations between the Q8P and BFP dimensions. The most prominent cross-framework pairings suggest possible areas of content correspondence, but the findings do not establish equivalence, convergent validity, or incremental validity of the Q8PS. The results should be interpreted as exploratory because the factorial validity of the Q8PS remains provisional.

The Q8PS should therefore be regarded as an emerging, culturally grounded descriptive instrument whose eight-domain scoring framework remains provisional. Further item refinement and validation against independent samples, validated comparison measures, and external behavioral criteria are required before broader applied use can be recommended.

However, this study had several limitations. First, the cross-sectional design limited our ability to infer causal relationships between the Q8P and BFP dimensions. Longitudinal or experimental designs are required to explore the developmental trajectories and causal mechanisms underlying these personality constructs.

Second, the sample predominantly comprised young adults from China, which may limit the generalizability of the results to other age groups, cultures, and clinical populations. Future studies should include participants from diverse demographic and cultural backgrounds to validate the cross-cultural applicability of the Q8PS.

Third, although the Q8PS demonstrated good internal consistency and test–retest reliability, further validation is needed in different contexts such as clinical settings or organizational environments to establish its broader utility and predictive validity.

Fourth, while innovative, the network analysis approach relies on partial correlations and may be sensitive to sample-specific variations. Replication of independent samples is necessary to confirm the stability and robustness of the identified network structure and bridge nodes.

Finally, the current study did not examine the behavioral or outcome correlates of the Q8P dimensions (e.g., mental health and job performance). Future research should explore how these culturally grounded traits predict real-world outcomes and enhance the practical relevance of the Q8PS.

## Data Availability

The original contributions presented in the study are included in the article/supplementary material, further inquiries can be directed to the corresponding author/s.
